# Understanding the Mechanism of Action of Melatonin, Which Induces ROS Production in Cancer Cells

**DOI:** 10.3390/antiox11081621

**Published:** 2022-08-20

**Authors:** Javier Florido, César Rodriguez-Santana, Laura Martinez-Ruiz, Alba López-Rodríguez, Darío Acuña-Castroviejo, Iryna Rusanova, Germaine Escames

**Affiliations:** 1 Biomedical Research Center, Health Sciences Technology Park, University of Granada, 18016 Granada, Spain; 2 Department of Physiology, Faculty of Medicine, University of Granada, 18016 Granada, Spain; 3 Centro de Investigación Biomédica en Red Fragilidad y Envejecimiento Saludable (CIBERFES), Ibs.Granada, San Cecilio University Hospital, 18016 Granada, Spain; 4 Department of Biochemistry and Molecular Biology, Faculty of Science, University of Granada, 18019 Granada, Spain

**Keywords:** melatonin, reactive oxygen species, apoptosis, mitochondria, cancer

## Abstract

Reactive oxygen species (ROS) constitute a group of highly reactive molecules that have evolved as regulators of important signaling pathways. In this context, tumor cells have an altered redox balance compared to normal cells, which can be targeted as an antitumoral therapy by ROS levels and by decreasing the capacity of the antioxidant system, leading to programmed cell death. Melatonin is of particular importance in the development of innovative cancer treatments due to its oncostatic impact and lack of adverse effects. Despite being widely recognized as a pro-oxidant molecule in tumor cells, the mechanism of action of melatonin remains unclear, which has hindered its use in clinical treatments. The current review aims to describe and clarify the proposed mechanism of action of melatonin inducing ROS production in cancer cells in order to propose future anti-neoplastic clinical applications.

## 1. Introduction

Cancer continues to be a dreadful disease despite the continuous efforts made to develop novel treatment modalities. According to the GLOBOCAN report published in 2020, 19.3 million new cancer cases were expected in 2020, with an estimated 10 million cancer mortalities. Furthermore, with the number of cancer cases expected to reach 28.4 million around the world in 2040 [1], it is necessary to develop innovative treatments and to investigate new therapeutic targets.

Reactive oxygen species (ROS), which are generally considered byproducts of oxygen consumption and cellular metabolism, are formed by the partial reduction of molecular oxygen [2]. Thus, tumor cells have an altered redox balance compared to normal cells, which can be targeted by antitumoral therapies by increasing ROS levels and by decreasing antioxidant system capacity, leading to apoptosis. This is the case for certain chemotherapeutic compounds that impact the induction of ROS production, resulting in irreparable damage and cell death [3].

The hormone melatonin (aMT; N-acetyl-5-methoxytryptamine) is synthesized by the pineal gland as well as by several types of tissues. It is also well-known that melatonin is produced by mitochondria [4] at higher concentrations than in other cellular compartment [5,6]. Melatonin, together with its metabolites, are not only involved in multiple cellular processes [7] but are also potent free radical scavengers and broad-spectrum antioxidants with evolutionarily conserved properties [8,9]. Melatonin is capable of reducing free radical damage by acting directly as a free radical scavenger and indirectly by stimulating antioxidant enzyme activity [10]. In addition, melatonin plays an effective role in maintaining mitochondrial homeostasis, which protects against oxidative damage [11,12,13,14,15]. However, despite its widely recognized antioxidant role in protecting normal cells against cytotoxicity and apoptosis, melatonin, which induces apoptosis in several types of cancer cells, has also been shown to have pro-oxidant effects [16].

Various groups have, for many years, reported that high concentrations of melatonin can promote ROS generation, leading to cell death in a variety of cancers [17,18,19,20,21,22]; this suggests that melatonin can act as both an antioxidant and pro-oxidant in human cell lines, depending on the concentration and treatment duration. Furthermore, numerous studies have shown that melatonin enhances the cytotoxic effects of chemotherapeutic drugs on cancer cells, depending on the dose [23], thus suggesting that melatonin increases their chemotherapeutic effect [24].

In conclusion, melatonin is an ideal candidate for use in innovative cancer therapies. However, unlike its antioxidant effect, the way in which melatonin produces ROS in tumoral cells remains unclear. Thus, the identification of signaling pathways and key molecules associated with the pro-oxidant effect of melatonin is extremely important with regard to the effectiveness of clinical anti-neoplastic therapies.

## 2. Involvement of Receptors in Melatonin’s Pro-Oxidant Activity

Melatonin appears to exert some of its effects in humans through the MT1 and MT2 membrane receptors [25] and also provides high-affinity binding for the nuclear receptors ROR/RZR [26]. In addition to regular receptors, melatonin binds other proteins such as calmodulin (CaM) and calreticuline (CALR) [27,28], which interact with melatonin at concentrations ranging from sub-nanomolar to millimolar in mammalian cells [29]. In addition, the level of melatonin to which these targets respond can have concentration ranges of over seven orders of magnitude [30]. In this context, it has been widely demonstrated that high levels of melatonin are necessary to induce ROS production in tumor cells [19,20,31,32], thus highlighting the independent impact of low-affinity melatonin targets such as MT1, MT2, and ROR receptors. This is confirmed by melatonin’s pro-oxidant effect, which is unaffected by the MT1/MT2 antagonist luzindole and is not elicited by melatonin analogues with high affinity for MT1/MT2 receptors [29].

In contrast, it has been suggested that melatonin’s pro-oxidant activity derives from binding to calmodulin, to which other enzymes, such as phospholipase A2 (PLA2), associated with oxidative stress, also bind [29].

**Figure 1:**
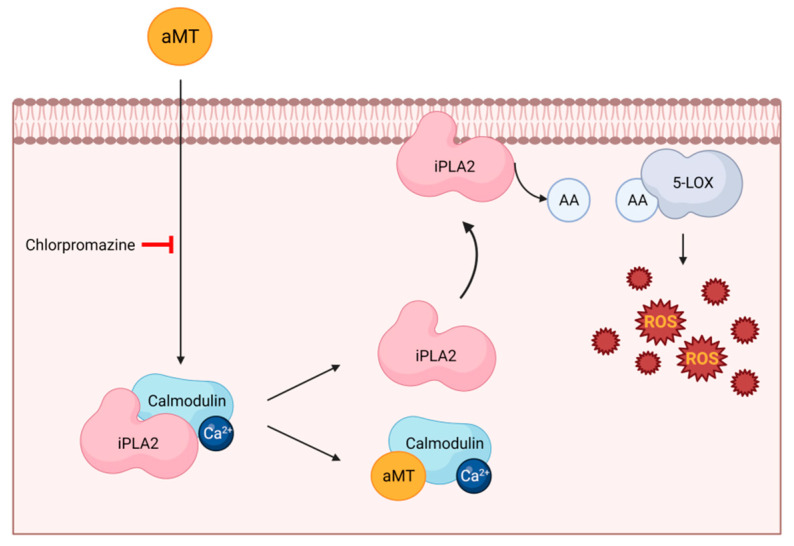
Melatonin induces ROS production in cancer cells through calmodulin binding. Melatonin binds to calmodulin, leading to the release of sequestered Ca^2+^-independent PLA2, which is then free to move to membranes and to release high doses of AA; in turn, liberated AA feeds 5-LOX to produce free radicals. Melatonin (aMT); Ca^2+^-independent PLA2 (iPLA2); arachidonic acid (AA); 5-lipoxygenase (5-LOX). Image created using BioRender.com(accessed on 16 July 2022).

The PLA2 enzyme cleaves membrane phospholipids, leading to the release of membrane-bound arachidonic acid (AA), which is processed by cyclooxygenases (COXs) and lipoxygenases (LOXs) to produce important inflammatory mediators, such as prostaglandins and leukotrienes, as well as to increase ROS production [33]. However, Ca^2+^-independent phospholipase PLA2 (iPLA2) can bind to calmodulin that is inactivated. Radogna et al. [34] demonstrated that melatonin, at high concentrations, binds to calmodulin, thus inducing the release of iPLA2 and, consequently, increasing ROS production in human tumor monocytes (U937 cells; Figure 1). The increase in ROS levels by melatonin is inhibited by chlorpromazine, which prevents melatonin from binding to calmodulin, which is insensitive to calmidazolium, which does not prevent this interaction [34].

Finally, iPLA2 and 5-LOX inhibitors also abolish melatonin’s ability to stimulate ROS production, indicating that these two enzymes are involved in melatonin’s pro-oxidant activity [34]. All these findings suggest that the binding of melatonin to calmodulin is required in order to induce ROS production.

## 3. Involvement of Molecular Pathways in Melatonin’s Pro-Oxidative Activity

In addition to its effect through its proven affinity for receptors, melatonin can induce oxidative stress directly by increasing ROS levels and indirectly by regulating the expression of various proteins involved in metabolic pathways, as described below.

### 3.1. The Sirtuin Pathway

Sirtuins, which are class III histone deacetylase enzymes, are key molecular proteins involved in oxidative stress [35] and play an important role in both normal and cancer cells [36].

Sirtuin-3 (SIRT3), which is located primarily in the mitochondrial matrix, regulates intracellular metabolism, mainly by modulating mitochondrial oxidative stress [37]. It also regulates superoxide dismutase 2 (SOD2) by deacetylating and activating the SOD2 transcription factor FOXO3a as well as by directly deacetylating and activating SOD2 dismutase activity [38]. Furthermore, decreased SIRT3 expression increases ROS-mediated oxidative damage, suggesting that SIRT3 inhibitors could be therapeutically beneficial [39]. Li et al. [40] demonstrated that melatonin potentiates the cytotoxic effects of shikonin (SHK) in HeLa cancer cells by inducing oxidative stress through the inhibition of SIRT3/SOD2 expression and activity. The combination of melatonin and SHK promotes apoptosis, which increases ROS production in various types of cancer cells. All these effects are reversed by ROS scavengers, thus suggesting that melatonin induces apoptosis in cancer cells by increasing ROS production. This is probably explained by the excessive levels of ROS induced by melatonin, which releases mitochondrial cytochrome C, leading to apoptosis in cancer cells [41].

**Figure 2:**
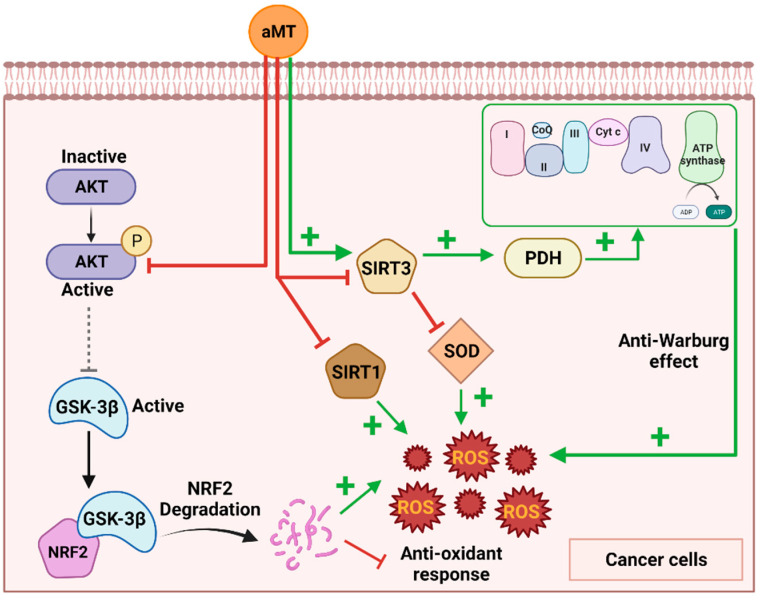
Different mechanisms by which melatonin induces ROS production in cancer cells. Melatonin inhibits the AKT pathway, leading to the activation of GSK-3β, which induces NRF2 degradation. On the other hand, melatonin regulates Sirtuin 3 (SIRT3) through its activation or inhibition, leading to an anti-Warburg effect or SOD inhibition, respectively. Finally, melatonin has been shown to inhibit SIRT1 in cancer cells. All these processes lead to an increase in ROS production and antitumor activity. Melatonin (aMT); glycogen synthase kinase-3β (GSK-3β); superoxide dismutase (SOD); pyruvate dehydrogenase (PDH). Image created using BioRender.com (accessed on 18 July 2022).

However, other authors, such as Chen et al. [42], have reported that melatonin increases SIRT3 activity in lung cancer cells. This results in the deacetylation of pyruvate dehydrogenase (PDH) to enhance complex I and IV activity, which increases ROS production and reverses the Warburg effect (Figure 2). Thus, melatonin induces cancer cellular apoptosis by elevating ROS generation due to an increase in OXPHOS through the stimulation of SIRT3 activity [42]. Further research is required to clarify melatonin’s mechanism of action via SIRT3.

SIRT-1, a conserved nicotinamide adenine dinucleotide (NAD+)-dependent deacetylase, is associated with melatonin’s antitumoral and pro-oxidative activity. Melatonin has been demonstrated to increase SIRT1 activity in normal cells, leading to a decrease in ROS production and the regulation of cell homeostasis [43,44]. However, SIRT1 overexpression in tumor cells correlates with the silencing of tumor suppressor genes and cancer resistance to chemotherapy. Melatonin in tumor cells, such as human osteosarcoma, has been shown to inhibit SIRT1, resulting in increased pro-oxidant and antitumor activity (Figure 2) [45]. The inhibition of SIRT1 through the use of the inhibitor sirtinol or SIRT1 siRNA also increases melatonin’s antitumor activity. The upregulation of SIRT-1 by its activator, SRT1720, attenuates melatonin’s antioxidant and antitumor activity, indicating that its induction of ROS production in tumor cells is activated by SIRT1 [45]. It has been suggested that melatonin is directly involved in controlling SIRT1, which inhibits its activity in cancer cells, in contrast to its stimulatory action in normal cells [46].

### 3.2. The Akt Pathway

Akt, which is a serine/threonine kinase that was previously known as protein kinase B (PKB), plays a crucial role in major cellular functions such as cell size, cell cycle progression, glucose metabolism regulation, genome stability, transcription, and protein synthesis. Akt promotes cell survival by mediating cellular growth factors and by blocking apoptosis through the inactivation of pro-apoptotic proteins as well as through the regulation of ROS balance [47]. A wide range of proteins are sensitive to phosphorylation by AKT, such as glycogen synthase kinase-3β (GSK-3β), a protein serine/threonine kinase involved in cell signaling that is phosphorylated on serine 9 and then inactivated [48].

Melatonin has been widely reported to modulate Akt in both tumoral and nontumoral cells [49,50,51,52]. In human melanoma cells (SK-MEL-1), Perdomo et al. [53] demonstrate that, as melatonin promotes GSK-3β dephosphorylation at serine 9 via Akt pathway inhibition, the activation of GSK-3β by melatonin results in an increase in ROS production (Figure 2). This was confirmed by the use of the GSK-3β inhibitor BIO, which partially abrogates the generation of ROS in response to melatonin. Perdomo et al. also reported that the pro-oxidative effects of GSK-3 are due to the degradation of NRF2, the master regulator of endogenous antioxidant responses [53]. This pattern was also reported in the glioblastoma cell line U87MG, where the inhibition of Akt triggers GSK-3β activity, which, in turn, switches off the antioxidant response of NRF2 [54].

### 3.3. Involvement of OXPHOS Induction in the Conflict between Melatonin’s Pro-Oxidative and Anti-WARBURG Activity

The Warburg effect, which is commonly associated with solid tumors, contributes significantly to their hardness, invasiveness, and metastatic capability as well as rendering them resistant to radio- and chemotherapies [55,56,57]. In most mammalian cells, mitochondria are an important source of hydrogen peroxide (H_2_O_2_) and superoxide anion (O_2_^−^) [58]. The Warburg effect of glycolysis upregulation in cancer cell energy metabolism could reduce the production of H_2_O_2_ and O_2_^−^ by decreasing OXPHOS activity [59]. Just as the inhibition of metabolic reprogramming induces ROS production in tumor cells, melatonin increases OXPHOS capacity and inhibits glycolysis in cancer cells, resulting in increased ROS production [20,42].

As shown in Figure 2, Chen et al. [42] reported that melatonin reverses the Warburg effect by stimulating the SIRT3/PDH axis in lung cancer cell lines. The pyruvate dehydrogenase complex (PDC) plays an important role in catalyzing the conversion of pyruvate to acetyl-CoA, which is associated with mitochondrial ATP production. Pyruvate dehydrogenase (PDH), which is the first and most important enzymic component of PDC, converts pyruvate to acetyl-CoA and then enters tricarboxylic acid (TCA) to produce ATP as well as electron donors such as NADH. In cancer cells, metabolic reprogramming results in the inhibition of PDH, leading to a decrease in acetyl-CoA. Pyruvate is then shunted away from the mitochondrial cancer cell metabolism through its reduction to lactate (Warburg effect) [60]. Chen et al. [42] showed that melatonin promotes SIRT3 expression and PDH deacetylation in order to enhance complex I and IV activity, leading to a reversal of the Warburg effect and ROS production, followed by cell death in lung cancer cells (A549, PC9, and LLC). This hypothesis was corroborated using 3-TYP, a selective SIRT3 inhibitor that abolishes melatonin’s ability to stimulate PDH activity and ROS production, suggesting that the melatonin regulation of SIRT3 is required to reverse the Warburg effect. Thus, not only does SIRT3 inhibit SOD, as described above, but it also induces PDH, which together lead to ROS production and apoptosis in tumoral cells [42].

Our research group found that treatment with melatonin inhibits metabolic reprogramming by inhibiting glycolysis and increasing OXPHOS activity, leading to the production of ROS in head and neck squamous cell carcinoma (HNSCC) [20]. Thus, enhanced OXPHOS activity increases the levels of ROS, thereby inducing cancer cell death [61]. In primary human kidney mesangial cells, melatonin can also induce rapid ROS generation via the antimycin-A-sensitive site in mitochondrial complex III [9].

It may also be possible that melatonin not only reverses Warburg effects, leading to ROS production in cancer cells, by activating sirtuins but also regulates the malignancy-promoting transcription factor hypoxia-inducible factor-1α (HIF-1α). Therefore, it is also suggested that the underlying mechanism of the pro-oxidant effect of melatonin on cancer cells involves the inhibition of HIF-1α by melatonin. HIF-1α is a key transcription agent involved in mediating Warburg-type metabolisms in diseased cells [62,63]. HIF-1α is also part of an oxygen sensing system that is activated when the partial pressure (pO_2_) of intracellular oxygen becomes depressed. HIF-1 plays a critical role in stimulating the mitochondrial pyruvate dehydrogenase kinase (PDK), which leads to the inactivation of the pyruvate dehydrogenase complex (PDC), thereby reducing the mitochondrial conversion of pyruvate to acetyl coenzyme A and stimulating the Warburg effect [64,65]. Research has demonstrated that melatonin, either directly or indirectly, inhibits HIF-1 in cancer cells [66,67,68], thus likely reversing the Warburg effect and inducing both ROS production and cell death. However, despite the proven close relationship between melatonin’s anti-Warburg effect and increased ROS production, further research is required to understand the precise mechanisms involved.

## 4. Decreased Antioxidant Defenses

The pro-oxidant and oncostatic effects of melatonin can be explained by the increased levels of intracellular ROS and by the decrease in antioxidant capacity exhibited in the melatonin-treated cells, as described above. Melatonin decreases antioxidant enzymes such as catalase, glutathione peroxidase (GSH-Px), and SOD and also increases lipid peroxidation in different cancer types (Table 1). However, in nontumoral cells, melatonin reduces oxidative stress damage to scavenging free radicals and increases antioxidant enzyme activity [8,69,70].

It has also been suggested that melatonin regulates antioxidant enzymes through the cellular prion protein (PrPC)-dependent pathway. Normal PrPC is a ubiquitous glycoprotein involved in various physiological cellular processes, including proliferation, differentiation, stress protection, and signal transduction regulation. PrPC is also involved in tumor resistance in colorectal cancer cells [74]. It also protects cells against oxidative stress by increasing the activity of antioxidant enzymes such as SOD and catalase [75], while the silencing of PrPC decreases this antioxidant activity [76,77].

Some studies have shown that melatonin reduces PrPC levels, leading to a decrease in antioxidant defenses in cancer cells [78]. Lee et al. [79] reported that PrPC levels increased in human oxaliplatin-resistant cell lines (SNU-C5/Oxal-R), resulting in an increased antioxidant effect through an increase in SOD and catalase activity. On the other hand, cotreatment with oxaliplatin and melatonin reduced the level of PrPC and consequently led to the suppression of antioxidant enzyme activity and increased superoxide anion generation in these cancer cells. The increase in superoxide anion levels is related to the activation of the endoplasmic reticulum (ER) stress-mediated signaling pathway and the induction of apoptosis through the regulation of apoptosis-associated proteins [79].

Melatonin’s induction of ROS and mitochondrial dysfunction via PrPC could be explained by the regulation of the expression of PTEN-induced putative kinase 1 (PINK1), a protein located in the outer mitochondrial membrane that maintains mitochondria homeostasis. Won Yun et al. [80] showed that melatonin suppresses PrPC and PINK1 expression, which leads to an increase in the production of mitochondrial superoxide in colorectal cancer cells. The impact of melatonin is greater when PrPC is silenced, indicating that the inhibition of PrPC expression enhances melatonin-mediated pro-oxidant activity [80].

## 5. Reverse Electron Transport (RET): Another Melatonin Mechanism That Could Induce ROS

In addition to the mechanism described above, melatonin may induce ROS production via reverse electron transport (RET). RET occurs in mitochondria when the pool of coenzyme Q becomes overly reduced by electrons from respiratory complex II or other enzymes as well as in the presence of high proton motive force (Δp). Under these conditions, CI activity reduces NAD+ to NADH with electrons from the ubiquinol pool, thus generating high levels of mtROS [81,82]. Complex I has also been found to produce ROS in a forward or reverse direction, depending on the substrates used to feed the respiratory chain, suggesting that a change in cell metabolism induces RET. We previously demonstrated that melatonin reverses metabolic reprogramming in HNSCC cells [20]. Given these findings and the melatonin-modified tumor metabolism, we determined whether melatonin increases mtROS via RET.

**Figure 3:**
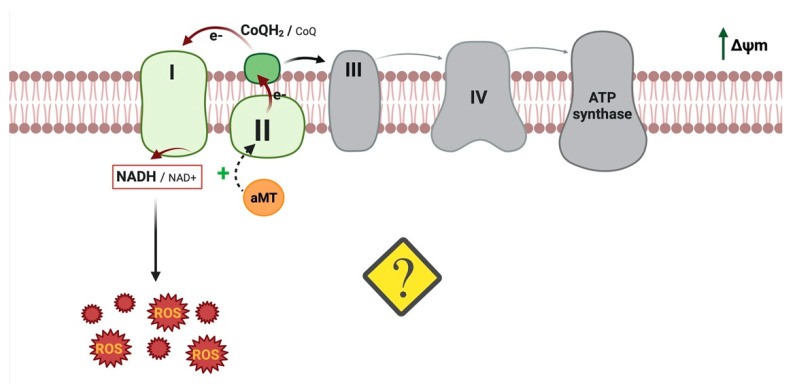
Possible mechanisms of action of melatonin to induce ROS production via RET. In our view, melatonin increases mitochondrial CII activity, membrane potential, and CoQH2/CoQ, leading to RET-ROS production. Image created using BioRender.com (accessed on 22 July 2022).

Thus, our research group reported [83] that melatonin increases mitochondrial CII activity, membrane potential, and CoQH2/CoQ, which are essential conditions for RET (Figure 3). Interestingly, mitochondrial complex inhibitors, including rotenone, which increases forward ROS production but decreases ROS production via RET, abolish the increase in ROS, indicating that melatonin increases ROS generation via RET. Under hypoxic conditions, HIF maintains ROS production as well as their integrity at physiologically low levels by decreasing respiratory activity [84]. However, it was demonstrated that melatonin has the ability to destabilize HIF-1α [85]. In previous research, we showed that melatonin inhibits tumor cells by reversing aerobic glycolysis [20], which is a key step in the destabilization of HIF-1α [86], either by suppressing synthesis or by promoting degradation [87]. We therefore hypothesize that the inhibition of HIF-1α is the principal regulator of melatonin’s pro-oxidant activity in cancer cells. Nevertheless, further research is required to elucidate melatonin’s mechanism of action, which induces ROS production via RET.

## 6. Debate around the Antioxidant Effects of Melatonin in Cancer Cells

The beneficial effects of melatonin’s antioxidant activity are widely documented in the literature. However, although the majority of studies described the pro-oxidant effects of melatonin on cancer cells, as detailed above, melatonin can also reduce ROS production in some cancer types.

The antioxidant activity of melatonin is due to its direct effect as a radical scavenger, increasing the expression and activity of antioxidant enzymes, and regulating mitochondrial homeostasis [88]. It has been suggested that factors such as treatment duration and molecular concentrations as well as target cell type and conditions affect the outcome of melatonin on oxidative stress [89]. Melatonin’s antioxidant activity has mainly been reported in three different states of tumorigenesis: cancer initiation, progression, and metastasis [90]. Prior to cellular malignancy, the antioxidant activity of melatonin maintains the genomic integrity of cells by preventing cellular tumorigenesis and by protecting DNA against oxidative damage. Melatonin prevents DNA mutations, either directly through free radical scavenging activity or indirectly through the inhibition of metal-induced DNA damage, by stimulating antioxidant enzymes, enhancing the DNA repair system, and suppressing pro-oxidative enzymes [91]. Once the oncostatic process has begun, some studies have reported that melatonin, at low doses, impairs the proliferation and apoptotic resistance of oral cancer cells by inactivating ROS-dependent Akt signaling, which is involved in the downregulation of cyclin D1, proliferating cell nuclear antigen (PCNA), and Bcl-2 as well as in Bax upregulation [92]. Interestingly, the antioxidative effects of melatonin are condition-dependent, as evidenced by the changing nature of its pro- and antioxidant activities in both in vitro cellular and acellular studies [88].

Other research has demonstrated that melatonin’s antioxidant activity has antiangiogenic and antimetastatic effects. Thus, melatonin has been reported to play an antiangiogenetic role in blocking ROS-activated extracellular regulated protein kinases (ERKs) and Ak pathways in oral cancers [92]. Melatonin also suppresses hypoxia-induced cancer cell migration and invasion through the inhibition of HIF-1α due to its antioxidant impact on hypoxia [68,93].

All these findings point to the dual impact of melatonin, depending on its regulation of ROS homeostasis [94]. Although one possible explanation for this dual effect is the differences in experimental procedures and cancer models used in the different studies, further research is required to better understand the contradictory activities of melatonin in cancer treatment.

## 7. Conclusions

The oncostatic effects of melatonin through its pro-oxidant actions have been widely described [17,18,19,20,21,22,95]. Its mechanism of action, however, remains unclear. This review described the possible pathways involved in ROS production by melatonin in cancer cells. These mechanisms include, besides melatonin receptors, sirtuins and Akt pathways and melatonin’s anti-Warburg activity. Furthermore, this review suggests that melatonin does not only induce ROS production by inducing oxidative stress but also by decreasing antioxidant defenses [19,20,23,31,40,71,72,73]. Finally, in view of new experimental data, we propose that melatonin induces ROS production in cancer cells, activating mitochondrial reverse electron transport [83]. Therefore, we describe and clarify a new mechanism of action of melatonin to induce ROS production in cancer cells, a finding that may be considered for its anti-neoplastic clinical applications.

## Figures and Tables

**Table 1:** Studies of melatonin’s effects on antioxidant defenses in cancer cells.

Type of Cancer Cell	Melatonin Dosage	Effects	Authors
Human colorectal cancer SW-480 cell line	300 µM	Decreased catalase and SOD activity	[71]
Human colorectal carcinoma HCT-116 cell line	10^−6^ M	Decreased catalase and GSH-Px activity and increased lipid peroxidation	[23]
Human histiocytic lymphoma U937 cell line	0.5, 1, and 2 mM	Decreased SOD2 activity	[40]
Human hepatocellular carcinoma HepG2 cell line	0 to 20 mmol/L	Decreased SOD2 activity	[72]
Human HNSCCCal-27 cell line	100, 500,and 1500 μM	Decreased SOD2 activity	[20]
Human HNSCC Cal-27 cell line	100, 500, and 1000 μM	Decreased GPx activity	[31]
Human HNSCC Cal-27 cell line	100, 500, 1000, and 1500 μM	IR + aMT at 100 μM: increased GPx activitya MT at 1000 μM alone or combined with CDDP: decreased GPx activity	[19]
Xenograft mouse colon cancer (CT26 cell line)	20 mg/kg	Melatonin improved SOD and GPx activity in nontargeted tissues and reduced these two enzymes in the tumor tissue.	[73]

## Abbreviations

5-LOX	5-lipoxygenase
AA	Arachidonic acid
aMT	Melatonin
CaM	Calmodulin
COX	Cyclooxygenases
ERK	Extracellular-regulated protein kinases
GSH-Px	Glutathione peroxidase
GSK-3β	Glycogen synthase kinase-3β
H_2_O_2_	Hydrogen peroxide
HNSCC	Head and neck squamous cell carcinoma
HIF-1α	Hypoxia-inducible factor-1α
iPLA2	Ca^2+^-independent phospholipase A2
LOX	Lipoxygenases
NAD	Nicotinamide adenine dinucleotide
O_2_^-^	Superoxide anion
PCNA	Proliferating cell nuclear antigen
PDC	Pyruvate dehydrogenase complex
PDH	Pyruvate dehydrogenase
PINK1	PTEN-induced putative kinase 1
PKB	Protein kinase B
PLA2	Phospholipase A2
PrPC	Cellular prion proteins
pO_2_	Oxygen partial pressure
RET	Reverse electron transport
ROS	Reactive oxygen species
SHK	Shikonin
SIRT3	Sirtuin-3
SOD2	Superoxide dismutase 2
